# The Phenomenon of Human Migration on the Breastfeeding Practices of Migrant Women: A Scoping Review

**DOI:** 10.7759/cureus.63614

**Published:** 2024-07-01

**Authors:** Lina María Murcia-Baquero, Elena Sandoval-Pinto, Christian H Guerrero, María de Lourdes López Flores, Erick Sierra-Diaz, Rosa Cremades

**Affiliations:** 1 University Center for Health Sciences, Department of Public Health, University of Guadalajara, Guadalajara, MEX; 2 University Center for Biological and Agricultural Sciences, Department of Cellular and Molecular Biology, University of Guadalajara, Guadalajara, MEX; 3 University Center for Health Sciences, Department of Microbiology and Pathology, University of Guadalajara, Guadalajara, MEX

**Keywords:** migrant women, emigration, exclusive breastfeeding practice, breastfeeding, human migration

## Abstract

Human migrations and different migratory flows have been as old as the practice of breastfeeding (BF). The reasons for migrating, the conditions, and its protagonists are so diverse, often constituting situations of vulnerability and risk for health decision-making at both the individual and collective levels. The relationship between BF and human migration is totally dynamic and includes multiple factors, which is why there is a need to characterize territorially its prevalence rate and variability depending on the context. The migration profiles that can be configured from factors, such as schooling, employment, the host country's health system, and support networks, among others, have heterogeneity between countries that make it necessary to identify them. This study is an in-depth review of the report on the practice of BF in migrant women. The Arksey and O'Malley method was used to perform the PubMed and SciELO searches. The search terms were "exclusive breastfeeding (EBF)," "breastfeeding," "migrant women," and "human migration," and original articles published in English, Spanish, and Portuguese were included. Of the 43 selected articles, differences were found between the various migrant groups, in variables such as socioeconomic level, education, access to health services, maternal knowledge, father factor, culture, and intention to breastfeed. The heterogeneity of the practice of BF between countries, as well as in intraregional migratory flows, establishes different protective or risk factors depending on where the phenomenon develops and its conditions.

## Introduction and background

The World Health Organization (WHO) recommends breastfeeding (BF) from the first hour of life to two years or more, due to the multiple benefits that it brings to both the child and the mother, such as reducing the risk of prevalent childhood diseases and reducing the risk of ovarian cancer, breast cancer, and postpartum depression in mothers, among others [1].

BF is a physiological act and a form of feeding that consists of the administration of breast milk from the mother to the neonate from birth to two years of age or more. It is also defined as a learned behavior and a biocultural phenomenon [2].

According to the report by the United Nations Children's Fund (UNICEF) and the World Health Organization (WHO), it is estimated that only 46% of boys and girls newborns worldwide are breastfed during the first hour of life. Data published in 2023 by the UNICEF indicate that, globally, only 48% of children continue to receive exclusive breastfeeding (EBF) up to five months of age [3].

The relationship between BF and human migration is dynamic, multifactorial, and poorly studied in the Latin American region, specifically after the development of one of the migration crises of the last decade. There are high degrees of uncertainty around the reasons, ways, and consequences of migrating for an individual, family, group, community, and country of origin and the receiving country, thus increasing even more burdens on a local health system [4,5].

According to the World Migration Report 2022 from the International Organization for Migration (IOM), at the end of 2020, the number of international migrants was estimated to be 281 million worldwide, increasing internal mobility due to secondary restrictions due to the COVID-19 pandemic. In addition, the Department of Economic and Social Affairs (DESA) in the migration data portal states that as of January 2021, 48.1% of the world population of international migrants are women [6].

Likewise, the differences in their practice are given principally by socioeconomic and cultural educational components, generating a significant impact on public health, which is why it is necessary to broaden our view of cultural and sociological behavioral guidelines.

The emerging health issues derived from these crises require a research approach and a characterization of the region in terms of the prevalence of BF. The objective of this article is to make a literary and conceptual review of the impact of the phenomenon of human migrations on the practice of BF in migrant women. BF is understood as a learned behavior and a biocultural phenomenon.

## Review

Materials and methods

Study Design

The methodology described by Arksey and O'Malley [7] was used. A question was defined for the literature review, and subsequently, an information search was initiated through PubMed and SciELO.

The study design is based on a systematic review of quantitative and qualitative studies that address sociodemographic data and different surveys of BF practice in migrant/immigrant women identified as the main group or comparative group. The definition of "migrant" established generically by the IOM was used [8]. The initial search concepts for the review are EBF, BF, and health, as well as human migration terms optimized by the MeSH tool, combined with the Boolean operators AND and OR. A search of the gray literature was carried out for information on the Latin American region, expanding with search terms to migrant women. In addition, related titles were manually searched in the reference list of the articles (Table 1).

**Table 1:** Keyword combinations.

Combination	Results	Selected articles
Breastfeeding AND immigrants	163	11
Breastfeeding exclusive AND immigrants	46	2
Breastfeeding AND emigrants	90	14
Breastfeeding exclusive AND emigrants	73	4
Human migration AND breastfeeding	50	4
Human migration AND breastfeeding exclusive	33	2
Breastfeeding AND female migration	87	2
Breastfeeding AND women migration	52	3
Total	594	42

Search Strategy

**Figure 1:**
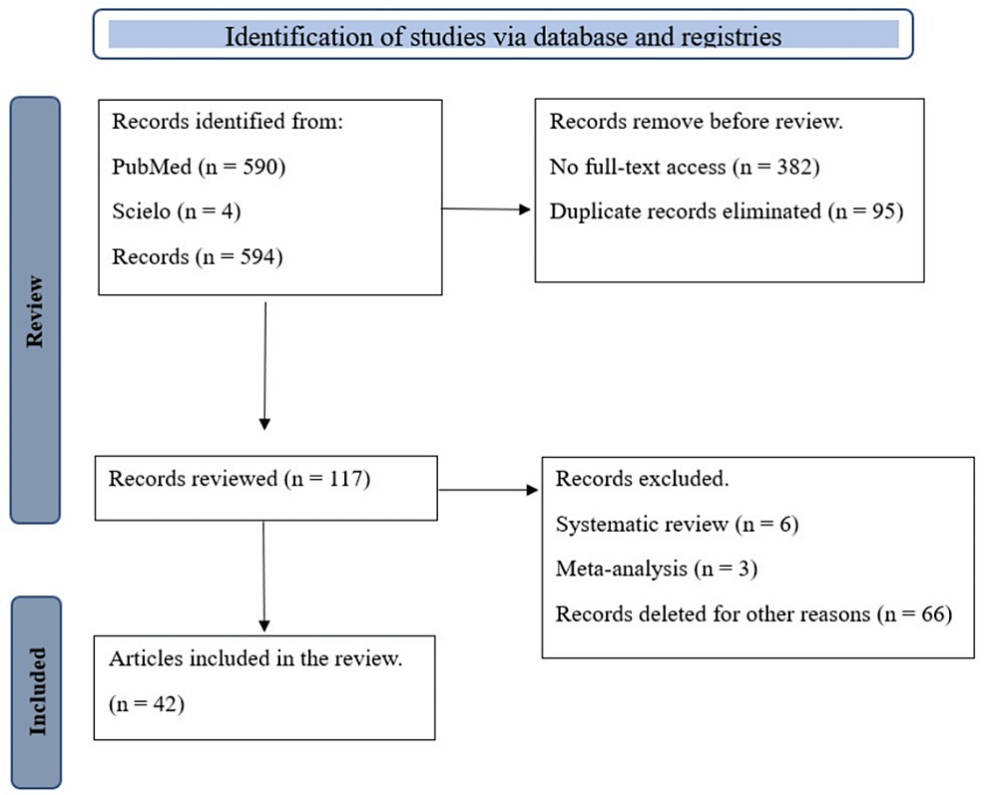
Preferred Reporting Items for Systematic Reviews and Meta-Analyses (PRISMA) diagram

Those with complete and/or free access to the document were initially selected, both in English, Spanish, or Portuguese, with a time range of 10 years, taking into account the historicity of the BF practice, such as human flows. For the purposes of this review, only one article dating from 1988 was taken into account in order to make visible the research purposes of the time. Subsequently, those articles whose title referred to other areas of knowledge were discarded and the duplicate articles were reviewed (Figure 1). The search was carried out in the period from May to August 2023 with a total of 42 references and complementary readings.

Inclusion and Exclusion Criteria

The main reference selection criteria were open access, quantitative, qualitative, and mixed studies, where at least one of the following variables was taken into consideration: population of migrant or immigrant women, which included a comparison of intraregional migration flows and between countries, and data on EBF and BF. The excluded works were systematic reviews and meta-analyses. All articles included were full texts. Reviews and manuscripts with only an abstract were excluded. The search, review, and selection of articles were carried out for four months (May-August 2023). A total of 42 references were considered for the review (Table 2).

**Table 2:** Studies included in the systematic review. EBF: exclusive breastfeeding, BF: breastfeeding

Author (year)	Region	Sample	Study design	Outcome
Yin et al. (2020) [9]	China	6.896	Cross-sectional	-EBF < 6 months -BF -Age-appropriate breastfeeding
Oves et al. (2014) [10]	Spain	1.452	Cohort	-EBF -Predominant BF
Thepha et al. (2018) [11]	USA	31	Qualitative	-BF
Tavoulari et al. (2015) [12]	Greece	428	Cohort	-EBF -Formula feeding -Partial BF
Grewal et al. (2016) [13]	Norway	187	Cross-sectional	-EBF -BF
Swigart et al. (2017) [14]	México	425	Mixed	-EBF -BF
Choudhry et al. (2012) [15]	UK	20	Qualitative	-EBF -BF -Formula feeding
Benjumea et al. (2011) [16]	Colombia	1.316	Cross-sectional	EBF BF
McLachlan et al. (2006) [17]	Australia	300	Cross-sectional	-EBF -Formula feeding
Walters et al. (2023) [18]	Uganda	63	Qualitative	-BF
Gibson et al (2006) [19]	USA	4.207	Cohort	-BF ever -BF 6 months
Lesorogol et al. (2018) [20]	Haití	589	Mixed	-EBF
Rojas et al. (2015) [21]	Venezuela	1.132	Cross-sectional	-BF
Zhou et al. (2020) [22]	Ireland	322	Mixed	-BF
Young et al. (1988) [23]	USA	158	Cross-sectional	-BF
Ravindranath et al. (2019) [24]	India	131	Mixed	-BF
Iglesias et al. (2021) [25]	Spain	19	Qualitative	-BF
Rodríguez et al. (2022) [26]	Chile	173	Cross-sectional	-EBF -BF
Rayment et al. (2016) [27]	London	57	Qualitative	-BF
Arora et al. (2017) [28]	Australia	1.035	Cohort	-BF ever
Lok et al. (2015) [29]	Hong Kong	2.827	Cross-sectional	-BF ever
Aguilar et al. (2019) [30]	Spain	73	Cross-sectional	-EBF -BF
Wu et al. (2015) [31]	Taiwán	21.217	Cohort	-BF ever -Predominant BF -BF ever
Harley et al. (2007) [32]	USA	499	Cohort	-BF started. -EBF at 4 months -BF at 6 months -BF per year
Henderson et al. (2018) [33]	UK	5.332	Cross-sectional	-EBF -EBF at 3 months
Kuswara et al. (2020) [34]	Australia	289	Cross-sectional	-EBF -EBF at 1 month -EBF at 3 months -EBF at 4 months -Any BF
Jessri et al. (2013) [35]	Canadá	22	Qualitative	-BF
Lisi et al. (2022) [36]	Brazil	31	Qualitative	-BF
Busck et al. (2014) [37]	Denmark	42.420	Cross-sectional	-BF at 4 months -Suboptimal BF
Bartsch et al. (2018) [38]	Canadá	1.044	Cohort	-EBF -EBF at 2 months -EBF at 4 months -EBF at 6 months
Brene et al. (2018) [39]	Berlín	6.884	Cohort	-BF
Yalçin et al. (2022) [40]	Turkey	76	Qualitative	-EBF -BF
Frenoy et al. (2021) [41]	Paris	456	Cross-sectional	-EBF -BF
Cinquetti et al. (2019) [42]	Italy	6.017	Cohort	-EBF -BF
Tulpule et al. (2022) [43]	Australia	1.011	Cross-sectional	-EBF -BF
Dennis et al. (2014) [44]	Canadá	1.184	Cohort	-EBF
Nolan et al. (2015) [45]	Ireland	9.700	Cohort	-BF
Hawkins et al. (2008) [46]	UK	6.478	Cohort	-BF
Zahra et al. (2022) [47]	Australia	522	Cross-sectional	-EBF -BF
Hohl et al. (2016) [48]	USA	20	Qualitative	-EBF -BF
Zhang et al. (2020) [49]	China	10.408	Cross-sectional	-EBF -Predominant BF
Kana et al. (2018) [50]	Portugal	7.065	Cohort	-EBF -Any BF

Results

The information collected was organized according to the frequency of the factors that interfered with the BF process and included data on the demographic profile of the migrant population and the use of confounding variables that statistically become relevant depending on the context and territoriality.

The information collected shows differences between the migrant and non-migrant populations regarding the practice of BF and EBF both between countries and in intraregional migratory flows [9,10]. These differences are the product of variables, such as socioeconomic level, education, access to health services, marital status, maternal knowledge, age, culture, and language, which indicates that a possible association between the migratory status of urban and rural areas can modify the practice of EBF and BF [9,11].

Main Factors for Abandoning EBF

Most studies evaluate the effect of migration on EBF due to its impact on the maintenance of BF practice and maternal and child health. The main reasons for abandoning EBF before six months were the perception of low production [9,12,13], use of bottles or formulas, consumption of other drinks for curative purposes [14,15], lack of satiety, and work factors [9,10].

Regarding the main maternal factors as the origin of abandonment, Rincón et al. identified in 653 women from different regions that the main causes of abandonment of BF were maternal health due to lack of production (34.8%) and the effect of illness or weakness (22.2%), lack of information (20.6%), or lack of motivation (1.4%) [16].

A study by Oves et al. reported that 69.5% of mothers of Spanish origin started BF at the time of hospital discharge compared to 75.4% of immigrant mothers (relative risk (RR) 0.74). They later indicate that 44.3% of mothers of Spanish origin maintained BF at three months compared to 53.8% in immigrant mothers [10].

Likewise, women of Vietnamese origin residing in Australia are 19% less likely to breastfeed exclusively during the early postpartum period [17]. Vietnamese women more frequently described sore nipples as their main problem (63%) compared to Australian women (17%) [12].

Of the aforementioned factors for abandoning BF within the migratory phenomenon, employment outside the home is decisive and considered a barrier to BF [18-20], since evidence postulates that the higher the mother's income, the less time of coexistence with the child, which makes BF difficult. Studies in the European population show that employed mothers are 36% less likely to practice EBF, in addition to abandoning BF within the first three to four months. In addition, the work factor increases the use of bottles and formulas by up to 15% [9,20,21].

However, Gibson et al. attribute that socioeconomic level is not completely linked to behaviors regarding BF practice. Their study carried out on Hispanic mothers shows a weak correlation with the social and economic factors [19]. The "Hispanic paradox” is reported as evidence, which is the phenomenon where the health status is better than what their socioeconomic status would indicate compared to other populations with a comparable socioeconomic status.

The variety of results infers behavioral paradoxes regarding economic factors in the migrant population, revealing the transversality with which this phenomenon must be addressed. Economic conditions and possibilities are not always going to be facilitating factors for the practice of BF.

Even providing breast milk in precarious economic conditions can favor the phenomenon of “last resort” whose only way to continue feeding the child is precisely by increasing the frequency or extending the time of offering breastmilk feedings [20].

BF Knowledge and Practices

Knowledge such as previous experiences in BF, benefits of the practice, techniques, and conservation, among others, was analyzed, with previous experience, age of the woman, and maternal education being decisive.

Rojas et al., in a study carried out on Venezuelan mothers from 18 different regions, concluded that 65% of them had a deficient level of knowledge [21], without taking into account previous experiences from previous pregnancies that could modify their results. On the other hand, Zhou et al. mentioned that multiparous mothers with previous experience in BF were more likely to initiate it compared to those who had never breastfed [22]. The difference between theory and practice are relevant findings that even transcend time [23,24].

Although most studies report knowledge of the multiple benefits of breastmilk and the advantages over formula milk and other foods [14,25,26], the literature reports the acceptance of fathers and mothers of providing other foods before six months, including cow's milk [18].

It should be noted that in the study by Mclaclan et al., Vietnamese women did not recognize the benefit of colostrum compared to milk produced days later [17] or did not offer it [13]. This lack of knowledge about colostrum can lead to incorporating other practices recommended by first-degree relatives, such as mothers and/or grandmothers [27].

Regarding education, a study carried out in Australia reports a negative association between the level of secondary education and the practice of BF [17], and the higher the educational level, the greater the difficulty for BF [18]. This is contrary to what was recorded in the CALINA study, where the high educational level of the native population benefited the practice [10]. Similarly, in Australia, with a diverse ethnic population, the higher the educational level and occupation of the mother, the greater the probability of starting BF [28]. A study conducted in Hong Kong with women of Chinese origin reports a 70% lower probability of BF with a low educational level [29].

However, other studies in the Chinese population suggest that those with a lower educational level have a greater probability of BF [30]. On the contrary, a study carried out in Taiwan on a group of migrant women does not report a significant association between educational level and the practice of BF [31].

Intention to Breastfeed

The intention to breastfeed in migrant and non-migrant women varies according to the region and previous experiences. Studies show important intentionality and planning regarding EBF up to six months of age [9]. Harley et al. reported that BF was initiated by 98% of women who said they intended to breastfeed, compared to 59% of women who said they did not intend to breastfeed [32]. There is a case study of Chinese women versus those of Spanish origin who were significantly less likely to initiate EBF after birth (adjusted for socioeconomic status (SES), parental education level, age, caesarean sections, and birth weight) (OR = 0.21; 95% CI: 0.05-0.91; p = 0.037) [23]. In another study on Vietnamese women, it was observed that they had the lowest level of initiation of BF compared to Australian women [17].

This intentionality could be categorized according to the migratory time, as reported in a study on the effect of time of residence in another country [32,33].

Health Systems

The formation and functioning of the different health systems in the world, as well as their main actors in the public and private spheres, are closely related to the practice of BF. In Mexico, a study reported that access to the different actors in local services and where the most information about BF is obtained from health clinics with 66.36%, doctors with 39.73%, nurses with 26.94%, and health promoters with 13.24% [14].

In the practice of BF, health professionals and nursing services are identified as support [9,34] or as a negative influence [25], promoting the supply of foods other than BF, corresponding to health personnel as the main supply of other foods (64.1%) [16]. Other studies report contradictory behaviors of health professionals, according to particular experiences and beliefs [35]. In Brazil, it is reported that three migrant women who were actively BF started a medical formula on medical advice [36]. Other studies do not report significant differences between the migrant and non-migrant populations regarding support at the hospital level for the practice of BF [37].

The different non-governmental organizations (NGOs) that are supporting migration processes, within possible deficiencies and/or limitations of local health systems, are identified as a possible facilitating factor of BF [18].

Migratory Profiles

Biological and socioeconomic variables analyzed in isolation do not explain the prevalence rate of BF among the different population groups, their behavior being diverse as the reason for migrating [19,38].

These migration patterns vary between countries and time periods and are mainly due to the maternal origin and their attitude toward BF [30,39]. The characteristics of these “new” patterns may even be risk factors for the practice of BF, as concluded by Yalcin et al., in their study with a Turkish population [40].

In countries such as Spain, China, the United Kingdom, southern Europe, Latin American countries, and African regions, migrant women maintain a higher prevalence rate than “native” women [12,13,26,41,42]. In general, nine out of 10 immigrant mothers breastfed compared to one or two non-migrant mothers [19,33,43]; however, studies carried out in Australia do not agree with the above.

Cohort studies carried out mainly at hospital discharge found that 69.5% of mothers of Spanish origin breastfed compared to 75.4% of immigrant mothers with a Spanish/immigrant (RR = 0.74), and at three months, 44.3% of mothers of Spanish origin maintained BF compared to 53.8% in immigrant mothers. These studies also allow for establishing migratory categories according to the time of migration and other variables that could interact with each other, achieving a local migratory profile [10,30,33,44,45].

These interactions change the behavioral references on the practice of BF being higher for five years or less and having a negative effect on the duration of residence for immigrants [32,40]. An additional year of living in the United States was associated with a 4% decrease in the odds of BF (OR = 0.958, p < 0.01) and a 3% decrease at six months (OR = 0.971, p < 0.05). A study in the United Kingdom reports a 5% lower probability of BF for every additional five years of residence in that country [46].

The possible exposure to the marketing of dairy formulas may be greater in these populations, as reported by Lisi et al., in their study with populations from Brazil and Portugal, through free samples or community or health participation gifts [36]. The imagination about the quality of dairy formulas produced in the host country and their social and cultural acceptance make their use preferable [22]. Ethnic origin can also increase the probability of using formula milk, as reported in a study carried out in Australia, with mothers of Vietnamese origin [47].

Parent Factor

The influence of the social and family factors is established negatively or positively in the decision to provide BF [17,20,48]. Harley et al. raised a limitation in the information on the father's attitudes and support for BF since the recording of the findings is generally covered by “couple,” “parents,” or “marital status” [49], which are important in the duration of the practice. Few investigations correlate paternal migratory status with the onset and/or maintenance of BF [14,17,45].

A study by Gibson et al. found that having a partner born in the United States decreased the odds of initiating BF by 83% (OR = 0.170, p < 0.01), and for each year a parent lived in the United States, a mother was 5% less likely to breastfeed (OR = 0.949, p < 0.01). Vietnamese women had the lowest rate of BF initiation (75%) and perceived their partners as more negative about BF [17].

Discussion

The practice of BF is still below the values ​​recommended by the WHO, although it has been widely studied and documented, which means a constant need to strengthen and consolidate its practice, mainly in emerging and temporary scenarios, such as migratory flows. Given the benefits of BF, any increase in its duration would have an impact on public health, so it is necessary to broaden our view to cultural, anthropological, behavioral, and sociological approaches.

The behavior of BF and maternal migratory status shows an important association, surpassing in several cases the concept of the "healthy migrant effect," deducing on the contrary that given the conditions of migration, their health conditions and their decisions regarding BF deteriorate more and more. The heterogeneity of the results presented is clear, as well as the different factors that can condition the practice of BF, with acculturation being observed as a risk factor. However, this behavior could present variability in the European context [50].

It is observed that immigration status is one of the most important conditions for the practice of BF in migrant populations. This aspect can be identified as a protective factor and a risk factor, as well as the invisibility of other derived circumstances that may lead to greater health inequity. Ethnic origin also reflects a variability of results that makes us understand territoriality [9,49]. Moreover, incorporating the ethnic origin of the father is considered a key actor for accompaniment and positive support for BF. Fathers can identify ways to support women [18].

The importance of differentiating the different nominal categories used in the reviewed studies (migrant, refugee, and asylum request), among others, which may occur depending on the country and local political situation, is highlighted [18].

It is noteworthy that the studies identified do not delve into possible adverse conditions of migratory transit (violence) that could impact the decision to breastfeed.

Regarding the years of residence in the host country, there seems to be a coupling and acceptance of health norms and practices, reconfiguring previous beliefs and customs, and prevailing already established hegemonic behaviors, the above can mainly mean a risk factor in countries where the prevalence of EBF and BF is low.

It is observed that other components emerge from the migration factor that can link the practice of BF, such as the labor and economic factors, seeing the complexity of linking BF to other contexts of daily life. Economic access can condition the use of milk formulas, where these formulas are often offered so that women have more time for both formal and informal work activities [25].

Overcoming the labor barrier could be one of the most complex in migratory contexts and vulnerable populations, where donations of commercial milk to migrant families are increasingly common in the context of humanitarian aid.

Regarding the main difficulties for BF, the perception of a small amount of milk and other physical factors of the mother (pain and nipple injuries) had a similar behavior in both migrant and non-migrant women. This is reported by both qualitative and quantitative studies, recognizing that maternal factors are predominant. Low-birth-weight babies did not significantly influence BF [29].

## Conclusions

The variability of the results and the identification of factors that influence the practice of BF, mainly sociocultural, establish the need to create a migrant profile according to context and territory. The acculturation process can play a protective or risky role depending on where the phenomenon develops, its conditions, and how different migrant groups relate to local health systems.

Cohort studies that established migratory categories defined as the maximum interaction time of six years may limit the information by presenting memory biases. The age ranges of the mothers were variable, which is a topic to be considered for future studies given the relevance and differentiation of pregnancies in adolescent ages, the most vulnerable group in the context of human movements.
